# Diabetic ketoacidosis presenting with atypical hemolytic uremic syndrome associated with a variant of complement factor B in an adult: a case report

**DOI:** 10.1186/s13256-016-0825-7

**Published:** 2016-02-24

**Authors:** Ziqiang Zhu, Hui Chen, Rupinder Gill, Jenchin Wang, Samuel Spitalewitz, Vladimir Gotlieb

**Affiliations:** 1 Department of Internal Medicine, Brookdale University Hospital and Medical Center, One Brookdale Plaza, Brooklyn, NY 11212 USA; 2 Division of Hematology/Oncology, Brookdale University Hospital and Medical Center, One Brookdale Plaza, Brooklyn, NY 11212 USA; 3 Division of Nephrology, Brookdale University Hospital and Medical Center, One Brookdale Plaza, Brooklyn, NY 11212 USA

**Keywords:** DKA, aHUS, Complement factor B, Variant

## Abstract

**Background:**

Non-Shiga toxin-associated hemolytic uremic syndrome is known to be caused by dysregulation of the alternative complement pathway. Infections, drugs, pregnancy, bone marrow transplantation, malignancy, and autoimmune disorders have all been reported to trigger episodes of atypical hemolytic uremic syndrome. To the best of our knowledge, there have been no previous reports of an association between diabetic ketoacidosis and atypical hemolytic uremic syndrome.

**Case presentation:**

We describe a case of a 26-year-old Spanish man who presented with diabetic ketoacidosis and was found to have the triad of microangiopathic hemolytic anemia, thrombocytopenia, and acute kidney injury. The patient had a normal ADAMTS13 (a disintegrin and metalloproteinase with a thrombospondin type 1 motif, member 13) activity level, and his renal biopsy demonstrated predominant changes of diabetic glomerulosclerosis with an area compatible with thrombotic microangiopathy suggestive of superimposed atypical hemolytic uremic syndrome. Complement sequencing subsequently revealed a potential causative mutation in exon 12 of complement factor B with changes of lysine at amino acid position 533 to an arginine (CFB p.K533R).

**Conclusions:**

To the best of our knowledge, this is the first case report of diabetic ketoacidosis presenting with atypical hemolytic uremic syndrome associated with a variant of complement factor B in an adult patient.

## Background

Hemolytic uremic syndrome (HUS) is characterized by the triad of microangiopathic hemolytic anemia, thrombocytopenia, and acute kidney injury. The majority of the cases are seen in childhood and are caused by Shiga-like toxin (so-called typical HUS). Non-Shiga toxin-associated HUS (atypical HUS, or aHUS) is known to be caused by dysregulation of the alternative complement pathway due to genetic mutations or neutralizing autoantibodies [1]. Infections, drugs, pregnancy, bone marrow transplantation, malignancy, and autoimmune disorders have all been reported to trigger episodes of aHUS [1]. To the best of our knowledge, there have been no reports of an association between diabetic ketoacidosis (DKA) and aHUS. We report a case of an adult patient with DKA presenting with aHUS associated with a variant of complement factor B (CFB).

## Case presentation

A 26-year-old Hispanic man with a history of type 1 diabetes that had been diagnosed between 12 and 14 years of age was brought to the emergency department (ED) after two episodes of new-onset seizures at home. A family member reported that the patient had stopped taking insulin for 1 day before presentation. He complained of epigastric pain and nonbloody, nonbilious vomiting at home. In the ED, the patient had another two episodes of tonic-clonic seizures.

His physical examination revealed that he was agitated, febrile (body temperature 38.5 °C), hypertensive (blood pressure 149/87 mmHg), and tachycardiac (heart rate 105 beats/minute). The remainder of his physical examination was unremarkable. His laboratory data (Table 1) were remarkable for hyperglycemia and high anion gap metabolic acidosis with an elevated β-hydroxybutyrate level compatible with DKA. No central nervous system pathology was revealed by a computed tomographic scan of his brain without intravenous contrast or by a lumbar puncture. The patient was admitted to the intensive care unit for management of DKA. His ketoacidosis resolved within 24 hours on intravenous fluids and an insulin drip. However, he continued to remain very drowsy in spite of correction of the DKA. Repeat laboratory data showed anemia (hemoglobin 9.1 g/dl, baseline value 11.8 g/dl 2 months prior), thrombocytopenia (150 × 10^9^/L, baseline value 416 × 10^9^/L 2 months prior), acute kidney injury with a blood urea nitrogen/creatinine ratio of 33/3.4 mg/dl (baseline value 40/1.4 mg/dl 2 months prior), and evidence of hemolysis (lactate dehydrogenase 1700 IU/L, indirect bilirubin 1.7 mg/dl) with schistocytes present on his peripheral blood smear. His presentation strongly suggested the possibility of thrombotic thrombocytopenic purpura/HUS, and emergent, empiric plasmapheresis was initiated while awaiting the result for the ADAMTS13 (a disintegrin and metalloproteinase with a thrombospondin type 1 motif, member 13) activity level. His additional serologic workup results, including complement components C3 and C4, antinuclear antibodies, antineutrophil cytoplasmic antibodies, cryoglobulins, anti-glomerular basement membrane antibody, and hepatitis B and C panels, were normal or negative.

**Table 1:** Hematological and chemistry laboratory values

Parameter (reference range)	Admission (day 0)	Postplasmapheresis (day 5)	Initiation of eculizumab (6 weeks after)
WBC, 10^9^/L	12	7.2	5.3
Hb, g/dl	9.1	8.2	8.0
Platelets, 10^9^/L	150	219	202
Serum Na^+^, mEq/L	142	135	130
Serum K^+^, mEq/L	4.4	5.5	5.6
Serum bicarbonate, mEq/L	7	26	27
BUN, mg/dl	33	34	66
Creatinine, mg/dl	3.4	3.6	5.6
Glucose, mg/dl	520	–	–
Indirect bilirubin, mg/dl (0.2–0.8)	1.7	0.7	–
LDH, IU/L	1700	763	–
Haptoglobin, mg/dl (43–212)	<15	–	–
pH	7.33	–	–
Anion gap	20	–	–
β-Hydroxybutyrate, mmol/L (0.02–0.27)	1.21	–	–
ADAMTS13	81 %	–	–
Schistocytes	Present	None	–
Urinalysis			
pH	6.0		
Protein, mg/dl	>300		
Glucose, mg/dl	>1000	–	–
RBC	25–30/HPF		
WBC	0–3/HPF		
Granular case	5–10/HPF		

*ADAMTS13* a disintegrin and metalloproteinase with a thrombospondin type 1 motif, member 13, *BUN* blood urea nitrogen, *Hb* hemoglobin, *HPF* high-power field, *LDH* lactate dehydrogenase, *RBC* red blood cells, *WBC* white blood cells

**Fig. 1:**
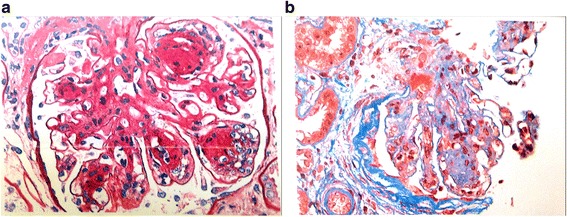
Kidney biopsy showed (**a**) moderate to marked, diffuse, and global increase in mesangial matrix-forming nodules compressing the capillary lumina and (**b**) only one glomerulus containing a fibrin thrombus involving the hilar region of the tuft

**Fig. 2:**
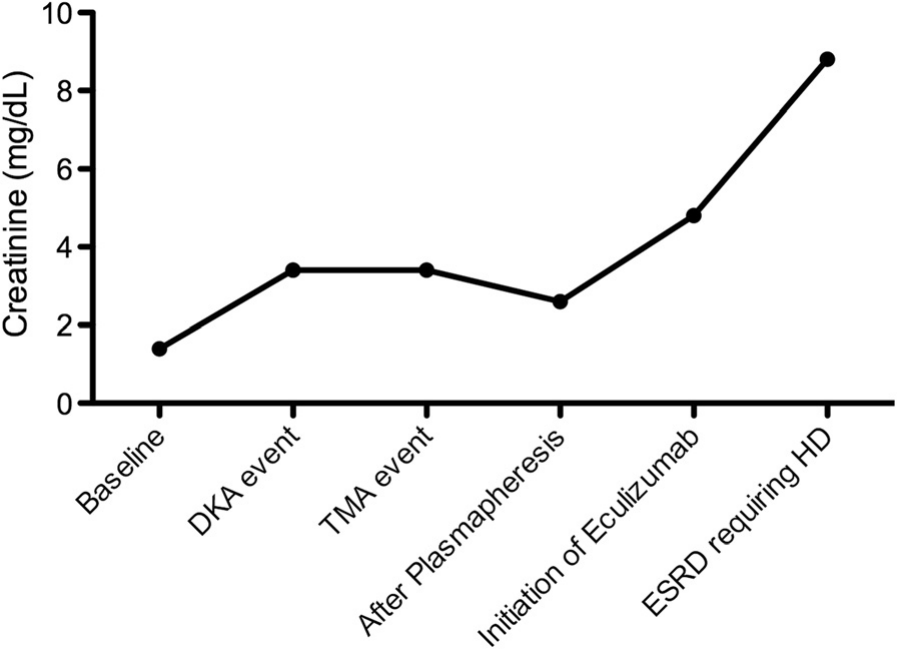
Serum creatinine changes during the course of hospitalization and follow-up. *DKA* diabetic ketoacidosis, *ESRD* end-stage renal disease, *HD* hemodialysis, *TMA* thrombotic microangiopathy

The patient responded with a dramatic improvement in mental status and hemolytic parameters after 5 days of plasmapheresis (Table 1). Unfortunately, his kidney function did not improve. On day 6, his ADAMTS13 activity was reported as normal (81 %, reference range 68–163 % activity). His anti-complement factor H (anti-CFH) antibody result was negative. His renal biopsy showed moderate to severe nodular diabetic glomerulosclerosis with superimposed thrombotic microangiopathy in a single glomerulus, suggestive of superimposed aHUS (Fig. 1). Complement sequencing of the coding regions of CFH, complement factor I (CFI), CFB, C3, membrane cofactor protein (MCP, CD46), diacylglycerol kinase ε, and thrombomodulin was completed. A heterozygous, nonsynonymous variant was identified in exon 12 of CFB with changes of a lysine at amino acid position 533 to an arginine (CFB p.K533R). The patient was started on treatment with eculizumab, a humanized monoclonal antibody targeting complement component C5, after he received meningococcal vaccine. He had no further episode of DKA or aHUS during 5 months of follow-up after initiation of eculizumab therapy. However, his renal function gradually deteriorated and hemodialysis was started (Table 1, Fig. 2). He is currently being evaluated for kidney transplant.

## Discussion

Extensive research has established an association between aHUS and the alternative complement pathway. It has been reported that mutations in the genes encoding proteins regulating the alternative complement pathway may result in abnormal activation of this pathway. It could be due to loss-of-function mutations in CFH, MCP, and CFI by either nonsense or missense mutations or by gain-of-function mutations in C3 and CFB [1]. Under normal circumstances, the *CFB* gene encodes the factor B protein, which is a component of C3 convertase, C3bBb, and catalyzes the proteolytic cleavage of C3 into C3a and C3b. Genetic mutations in the *CFB* gene may cause either enhanced formation of C3 convertase or increase its resistance to inactivation, thus leading to uncontrolled hyperactivity of the alternative complement pathway. *CFB* mutations have previously been reported to be associated with aHUS at a frequency as high as 1.4 % [2]. Table 2 lists all the previously reported cases with *CFB* mutations in patients with aHUS; the majority of these reported patients were children. The first two mutations in *CFB* gene F286L in exon 6 and K323E in exon 7 are both gain-of-function mutations that enhance the formation of C3 convertase [3, 4]. The *CFB* mutation (p.Lys350Asn) reported by Funato and colleagues causes resistance to decay acceleration factor and therefore increases C3bBb stability [5].

**Table 2:** Previously reported *CFB* mutations in patients with atypical hemolytic uremic syndrome reported in the English-language literature

Case reports	Sex	Age	Mutation; amino acid change
Fremeaus-Bacchi *et al.* [2]	–	–	p. V455I
Goicoechea de Jorge *et al.* [3]	M	23 years	c.858C>G; p.F286L
	M	4 months	c.967A>G; p.K323E
Tawadrous *et al.* [4]	F	8 years	c.1598A>G; p.K533R
Funato *et al.* [5]	F/M/F	8/6/20 years	c.1050G>C; p.K350N
Noris *et al.* [6]	–	–	R183W
Marinozzi *et al.* [7]	–	–	c.1598A>G; K508R
Bekassy *et al.* [9]	F	12 years	c. 1298T>C; c.L433S
Gilbert *et al.* [10]	F	4 months	c.967A>C; p. K323Q
Maga *et al.* [11]	–	–	c.497C>T; p.S166P c.608G>A; p.R203Q c.724A>C; p.I242L c.967A>C; p.K323Q c.1365C>T; p.M458I c.1598A>G; p.K533R
Roumenina *et al.* [19]	M	53 years	c.837A>C: p.D254G
	F	33 years	c.1050G>C: p. K325N
	F	19 months	c.1050G>C; p.K350N
Zhu *et al.*[present report]	M	26	p.K533R

Regarding our patient with adult-onset aHUS, we report the p.K533R mutation in exon 12 of *CFB* with change of a lysine at amino acid position 533 to an arginine. This mutation was previously identified in an 8-year-girl with aHUS reported by Tawadrous and colleagues [4], who believed it to contribute to the pathogenesis of aHUS. Further evidence in favor of the potentially pathogenic effect is the fairly rare minor allele frequency of 0.2 % in the Latino population, in which aHUS is a rare event [6]. More recently, Marinozzi and colleagues [7] performed comprehensive assessment of the p.K508R mutation, a more mature protein of p.K533R after removing the leader peptide, which leads to altered numbering for the same variant. They concluded that p.K508R most likely represents a rare benign polymorphism. Although they acknowledged that the p.K508R variant did confer moderately increased serine protease activity *in vitro*, they judged it as inadequate to explain the reduction of C3 clinically. On the basis of the American College of Medical Genetics and Genomics standards [8], current understanding of the clinical interpretation for this variant is that it is “a variant of uncertain clinical significance.” However, controversies still exist. Therefore, additional studies are required for further clarification of the contribution of this rare variant/polymorphism to the pathogenesis of aHUS. In addition, with new technology, more genetic mutations that contribute to the pathogenesis of aHUS are likely to be identified in the future [9–11]. Therefore, we speculate that the combination of multiple incompletely penetrant variants or a “trigger event” may contribute to the development of aHUS in some cases such as our patient’s.

It has been proposed that a “trigger event” or a “second hit” is related to precipitation of an episode of aHUS in a susceptible individual. These individuals may have gene mutations or antibodies to complement proteins that lead to uncontrolled continuous activation of the alternative pathway, resulting in the formation of the membrane attack complex. Various trigger events associated with aHUS have been reported previously, including infections, drugs, malignancy, pregnancy, or autoimmune diseases such as systemic lupus erythematous, C3 nephritic factor, and anticardiolipin. No obvious infectious etiology was identified in our patient. He was noncompliant with insulin therapy, which may lead to development of DKA. Subsequently, DKA may have been the “trigger event” for the episode of aHUS due to genetic predisposition with the CFB p.K533R variant. However, the possibility that aHUS may have precipitated DKA in our patient cannot be completely ruled out. Studies have shown that hemostatic changes leading to a thrombotic tendency occur during ketoacidosis [12, 13]. In addition, DKA elicits systemic inflammation associated with dysregulation of adhesion molecule expression and cytokine release by endothelial cells [14]. We propose that, in our patient, endothelial cell dysfunction during DKA enhanced the abnormality of endothelial damage and microvascular thrombosis mediated by his abnormal complement activity and further activated the complement pathway, leading to his aHUS episode.

Treatment of aHUS has been evolving rapidly with the recent approval of eculizumab, a humanized monoclonal antibody that binds with high affinity to the human C5 protein [15, 16]. End-stage renal disease or death was reported to occur in up to 65 % of patients with aHUS before eculizumab was introduced [17]. Eculizumab has been reported to be effective, with improvement of renal function as well as decreased episodes of aHUS. Our patient’s kidney function gradually worsened despite eculizumab therapy, which was likely secondary to progression of diabetic nephropathy because only a small area of superimposed thrombotic microangiopathy (TMA) was identified together with moderate to severe nodular diabetic glomerulosclerosis in the kidney biopsy and no further episode of aHUS has occurred since then. He was started on hemodialysis and currently is awaiting evaluation for kidney transplant. The patient will require standard maintenance treatment with lifelong eculizumab because only very limited evidence regarding discontinuation of eculizumab has been reported to date [18].

## Conclusions

To the best of our knowledge, we report the first case of DKA presenting with aHUS in an adult patient. We report a potential causative mutation for aHUS: p.K533R in exon 12 of *CFB*.

## Consent

Written informed consent was obtained from the patient for publication of this case report and any accompany images. A copy of the written consent is available for review by the Editor-in-Chief of this journal.

## Abbreviations

ADAMTS13: a disintegrin and metalloproteinase with a thrombospondin type 1 motif, member 13
aHUS: atypical hemolytic uremic syndrome
BUN: blood urea nitrogen
C3: complement component C3
C4: complement component C4
C5: complement component C5
CFB: complement factor B
CFH: complement factor H
CFI: complement factor I
DKA: diabetic ketoacidosis
ED: emergency department
ESRD: end-stage renal disease
HD: hemodialysis
Hb: hemoglobin
HPF: high-power field
LDH: lactate dehydrogenase
MCP: membrane cofactor protein
RBC: red blood cells
TMA: thrombotic microangiopathy
WBC: white blood cells
